# Efficacy and safety of a novel antiviral herbal preparation in ICU-admitted patients with COVID-19: A phase III double-blinded controlled trial

**DOI:** 10.22038/AJP.2023.23259

**Published:** 2024

**Affiliations:** 1 *Department of Community Medicine, School of Medicine, Shiraz University of Medical Sciences, Shiraz, Iran*; 2 *Shimi Teb Salamat Co., Shiraz, Iran*; 3 *Biotechnology Research Center, Pharmaceutical Technology Institute, Mashhad University of Medical Sciences, Mashhad, Iran *; 4 *Applied Biomedical Research Center, Mashhad University of Medical Sciences, Mashhad, Iran*; 5 *Ali Asghar Hospital, Shiraz University of Medical Sciences, Shiraz, Iran*; 6 *Department of Biostatistics, Shiraz University of Medical Sciences, Shiraz, Iran *; 7 *Shiraz HIV/AIDS Research Center, Institute of Health, Shiraz University of Medical Sciences, Shiraz, Iran *; 8 *Department of Medical Biotechnology, School of Advanced Technologies in Medicine, Shahid Beheshti University of Medical Sciences, Tehran, Iran*; 9 *Behbahan Faculty of Medical Sciences, Behbahan, Iran*

**Keywords:** Coronavirus, SARS-CoV-2, COVID-19, Treatment, ICU hospitalization, Mortality rate

## Abstract

**Objective:**

Despite an increasing number of studies, there is as yet no definite treatment developed for the coronavirus disease 2019 (COVID-19). In this clinical trial, we examined the efficacy of a novel herbal antiviral preparation in critically ill COVID-19 patients.

**Materials and Methods:**

A total number of 120 ICU-admitted patients with a diagnosis of COVID-19 pneumonia were recruited to the trial. Participants were equally randomized to receive either the novel antiviral preparation sublingually, for up to two consecutive weeks or till discharge, or placebo. Clinical and laboratory parameters as well as survival rates were compared between the two groups.

**Results:**

The cumulative incidence of death throughout the study period was 8.33% in the intervention group and 60% in the placebo group (risk ratio: 0.14; 95% confidence interval [CI], 0.05 to 0.32; p<0.001). On day 7, several parameters including white blood cells (WBCs) count, C-reactive protein, and SpO_2_ were improved for the treatment group compared with the placebo group (p-values of 0.05, 0.01, and <0.001, respectively).

**Conclusion:**

This preparation might be suggested as a potentially promising COVID-19 treatment.

## Introduction

The first cases of novel coronavirus disease (COVID-19) pandemic were initially reported in Wuhan city in the Hubei province of China during late December 2019 (Wang et al., 2020). Affecting billions of people globally, the severe acute respiratory syndrome coronavirus 2 (SARS-CoV-2) is known to be highly transmitted through respiratory fluids including droplets and aerosol particles with higher transmission rates in asymptomatic infected individuals, which has made the viral infection more prevalent and control of the disease more challenging (Hu et al., 2021). COVID-19 also demonstrates high morbidity among affected patients with a broader age range and despite lower proportion of pediatric patients, severe disease has been reported in COVID-19-affected children (Piroth et al., 2021). The majority of COVID-19 patients recover from the disease without receiving medical care or hospitalization and receiving supportive treatments, while a proportion develop severe grades of the disease requiring mechanical ventilation and hospitalization in the intensive care unit (ICU). The highest mortality rates among COVID-19 cases have been reported in 50-67% of these critically ill patients (Arentz et al., 2020; Myers et al., 2020). These patients develop severe complications of COVID-19 like acute respiratory distress syndrome (ARDS), which along with extrapulmonary conditions are responsible for poor outcomes (Oliveira et al., 2021). Prolonged ICU hospitalization and invasive mechanical ventilation cause pulmonary infections leading to septic shock and multi-organ failure as the immediate contributing conditions to the majority of deaths (Elezkurtaj et al., 2021). Thus, management of ICU-admitted patients has faced numerous challenges since the pandemic emergence. 

Theoretically, several steps of viral life cycle and SARS-CoV-2-encoded proteins (e.g. viral proteases) could be inhibited to block the virus pathogenesis (Bayat et al., 2021). However, despite a large number of studies, an efficient prophylactic or therapeutic regimen is yet to be discovered. Although several therapeutic approaches and supportive drug candidates, such as extracorporeal membrane oxygenation, anticoagulants are recommended as supportive therapies (MacLaren et al., 2022). Considering the paucity of effective therapeutic agents, especially in critically ill patients, this study examined the efficacy of an herbal antiviral preparation on the clinical symptoms, paraclinical parameters, and survival rates of ICU-admitted patients in a phase III clinical trial. This novel preparation is composed of several herbal ingredients including *Zataria multiflora *Boiss, *Glycyrrhiza glabra*, *Cinnamomum* *Vermont*, *Allium sativum *L., and *Syzygium aromaticum* extracts. The preparation has previously shown antiviral activity in cell experiments (unpublished data) and the current study is the first clinical experiment to investigate its efficacy in patients with COVID-19. Since mortality among ICU-admitted patients is significantly higher due to underlying conditions and medications, we set out to evaluate the efficacy of this antiviral preparation in critically ill COVID-19 patients.

## Materials and Methods

**Trial design and participants**

COVID-19 patients hospitalized in an ICU department of Shiraz University of Medical Sciences, Shiraz, Iran, were enrolled in this randomized, double-blind, placebo-controlled phase III clinical trial conducted during March 21- June 19, 2021. The trial was registered on the Iranian registry of clinical trials (https://www.irct.ir) (Trial registration: IRCT20200509047373N2. Registered on 13 March 2021). The trial site was at Ali Asghar hospital, as one of the ICU centers specialized for caring COVID-19 patients in Shiraz city, to enroll the eligible subjects. ICU-admitted patients were included in the trial with several eligibility criteria including having confirmed diagnosis of COVID-19, high pulmonary involvement in imaging, requirement to respiratory support but not mechanical ventilation (however, they may require mechanical ventilation during trial), aged 15 years or older, filled informed consent, and not participated in other clinical trials. Pregnant women were not entered to the study. COVID-19 definite diagnosis was made by the infectious diseases specialist according to the national COVID-19 committee’s guidelines for diagnosis and management of COVID-19 patients *via* SARS-CoV-2 reverse transcriptase polymerase chain reaction (RT-PCR) positive results or through characteristic radiographic presentations in chest computed tomography (CT) or radiographic imaging. Written informed consent was obtained from all patients or their legal representatives. The research was approved by the Ethics Committee of the Shiraz University of Medical Sciences (registration No. IR.SUMS.REC.1399.1367). Eligible patients were randomly assigned in a 1:1 ratio to either medication or placebo group. 

**Interventions**

Both groups received COVID-19 standard treatments including remdesivir and glucocorticoids according to the guidelines. Patients with underlying conditions also received their medications according to their past prescriptions. Patients assigned to the medication group received the antiviral preparation. Each patient was administered with 1 ml (containing 433 mg of active materials) antiviral preparation in drop form using dropper every 3 hour for up to two consecutive weeks. Moreover, patients who underwent mechanical ventilation received the medicine for up to 14 days or till discharge. Subjects in the placebo group were given a matching placebo containing normal saline in the same packaging with similar appearance, volume, and dosing as the active compound to maintain blinding. Standard of care was provided for all patients in both groups. 

**Antiviral preparation composition and dosing**

The novel antiviral preparation tested in this study has already been identified with antiviral properties in cell culture experiments (data not published), and comprises several active herbal ingredients. Plant materials included *Zataria multiflora* Boiss (5 mg), *Glycyrrhiza glabra* (10 mg), *Cinnamomum* *vermont* (2 mg), *Allium sativum *L. (2 mg), and *Syzygium aromaticum* (1 mg). Plant materials were purchased from a local botanical supplier in Iran, and were then approved by a plant classification specialist in the Department of Botany, Shiraz University of Medical Sciences, Shiraz, Iran. All plant materials were cleaned, dried, mechanically powdered, and extracted with 100% deionized water. The water was then evaporated using hot plate at 270°C to prepare the extract, followed by filtration. Plant material powders were mixed separately in 200 ml of distilled water. These suspensions were filtered by high-speed centrifuge and then mixed using a magnetic stirrer. The final extract was placed in the refrigerator until it was ready for use. The extract was formulated in the form of oral drop at a final concentration of 433±5 mg/ml to be used sublingually. The sterility testing was performed to check bacterial contamination in each extract. For sterility testing, nutrient agar plates were used and 0.1 mL of each extract was poured on them and incubated at 37°C. The bacterial growth was observed after 24 hr. Reverse phase chromatographic analyses were carried out under gradient conditions using C18 column (4.6 mm × 250 mm) packed with 5 μm diameter particles. The extract was dissolved in HPLC grade solvent. Then, the sample was sonicated using ultrasonicator for 10 min. The extract was filtered and injected to the HPLC column using a mobile phase comprising acetonitrile: 0.1% phosphoric acid mixture in a 30:70 ratio (v/v). The preparation was standardized based on 5.82 mg glycyrrhizic acid, and 2.5 µg cinnamaldehyde per each gram of the final preparation. The above procedures were undertaken in the Shimi Teb Salamat Co., Shiraz, Iran.

**Objectives**

The main objectives of this study were evaluating the impact of the antiviral preparation on the improvement of clinical parameters among ICU-admitted COVID-19 patients.

**Outcomes**

The main primary outcome was the COVID-19-associated death which was assessed on day 14 after enrollment. Other outcomes were clinical status, and changes in vital signs and laboratory parameters. Any possible adverse effect causing discontinuation of the study and impacting progression of the disease, as well as clinical or laboratory parameters were all recorded. 

**Sample size**

No prior data was available on the efficacy of the antiviral preparation in the management of COVID-19, so we performed a pilot study with 30 participants (15 per group) with the same protocol on similar patients and recorded the death rate as the preliminary data on the clinical efficacy of the preparation. Using the death rates in this study (P1 = 20%, P2 = 47%), by considering 90% power and the alpha level of 5%, we estimated a population size of 120 patients (60 per group). The sample size was calculated using two proportions formula. 

$n_{1}=n_{2}=\frac{(p_{1}\left(1-p_{1}\right)+p_{2}\left(1-p_{2}\right)){\left(Z_{1-\frac{\alpha }{2}}+Z_{1-\beta }\right)}^{2}}{{\left(p_{1}-p_{2}\right)}^{2}}$

According to the results, three (20%) of the participants in the antiviral preparation group and seven (46.7%) in the placebo group had an event (death through day 14). By considering 90% power to detect a between-group difference and the alpha level of 5%, we estimated a population size of 120 patients (60 per group).

**Randomization and masking**

Randomization was conducted by the statistical analyst using the website Randomization.com (http://www.randomization.com) and the permuted block randomization method with a block size of 4. Patients were enrolled by a health worker out of the research team and the statistical analyst performed patients’ allocation. Allocation was masked to all individuals including patients, physicians and statistical analysts. 

**Implementation**

Throughout the trial (day 0 to 14), patients were assessed for their clinical status. Any adverse effect or exacerbation in outcomes was recorded. Demographic characteristics, age, sex, BMI, and underlying diseases were collected at baseline. Several clinical and paraclinical parameters were evaluated in both groups on day 0, and for those survived on days 7 and 14. Vital signs including body temperature, blood pressure, respiratory rate, and blood oxygen saturation (SpO_2_), laboratory parameters such as hemoglobin (Hb) values, white blood cells (WBCs) count, and C-reactive protein (CRP) levels were measured. SARS-CoV RT-PCR was conducted at the first time point *via* standard mid-nasal sterile swab samples. Pulmonary involvement was investigated via chest CT-scans. Co-existing conditions were also compared between medication and placebo groups. No change was applied in the final protocol compared to the initial planned protocol. 

**Statistical methods**

The retrieved data was exported to SPSS software, version 18.0 (SPSS Statistics). To compare the qualitative and quantitative variables, Chi-square, independent samples t-test, paired samples t-test, and analysis of covariance (ANCOVA) were used and for non-normal variables, the non-parametric Mann-Whitney U test and Wilcoxon signed-rank test were employed. Binary logistic regression was also used to estimate the odds ratio (OR) of death. Survival analysis was conducted using the Kaplan-Meier curve and Cox regression.

## Results

**Figure 1:**
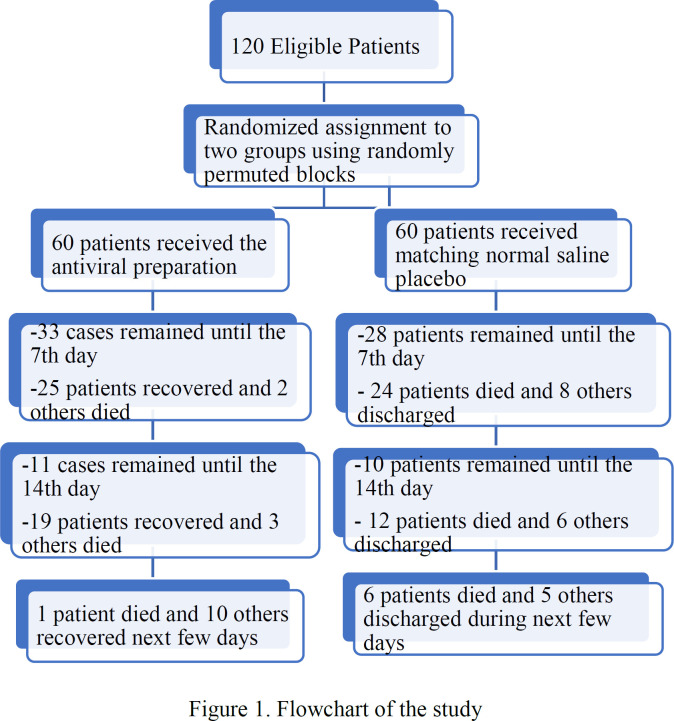
Flowchart of the study

A total of 120 patients were recruited to the study. Patients were all confirmed with COVID-19 and hospitalized to ICU department for receiving intensive care. Advanced pulmonary involvement and requirement to respiratory support via positive pressure non-invasive ventilation were reported for all patients. Among included participants, 68 patients (56.7%) were male and 52 patients (43.3%) were female. Mean age was 60.29*±*12.94 years with a range of 29-97 years (Table 1). Co-existing conditions, like cardiovascular disease, diabetes, or chronic respiratory disease were seen for 84 patients (70%) (Table 1). Sixty patients were randomly assigned to the medication group and an equal number was assigned to the placebo group. None of the patients received any vaccine before or during the trial. All patients completed the study, either recovered or died, and no participant left the trial (Figure 1). Table 1 shows some baseline information for both groups on day 0. Most of the parameters were comparable between the groups, however, mean age was different between the groups. Therefore, in order to adjust the age effect as a confounding variable, we used analysis of covariance (ANCOVA) test to compare the groups on the 7th day.

**Table 1:** Baseline clinical and laboratory values for medication and placebo groups

Qualitative variables						
Variables (unit)		Medication (n=60)	Placebo (n=60)		Total (n=120)	p-value ^a^
Age group (years)	<45	10 (17.2)	4 (6.8)		14 (11.7)	0.029
	45-64	33 (56.9)	27 (45.8)		60 (50)	
	≥ 65	15 (25.9)	28 (47.5)		43 (35.8)	
Sex (%)	Male	32 (53.3)	36 (60)		68 (56.7)	0.46
	Female	28 (46.7)	24 (40)		52(43.3)	
Disease history (%)	Yes	41 (68.3)	43 (71.7)		84 (70)	0.69
	No	19 (31.7)	17 (28.3)		36 (30)	
Co-existing Diseases	Cardiovascular disease	18 (30)	19 (31.7)		37 (30.8)	0.88
	Diabetes	11 (18.3)	9 (15)		20 (16.7)	
	Respiratory disease	3 (5)	4 (4)		7 (5.8)	
	Endocrine disease	4 (6.7)	2 (3.3)		6 (5)	
	Transplantation	3 (5)	1 (1.7)		4 (3.3)	
	Other	4 (6.7)	6 (10)		10 (8.3)	
	None	17 (28.3)	19 (31.7)		36 (30)	--
RT-PCR positive results	Yes	59 (98.3)	53 (88.3)		112 (93.3)	0.06
	No	1 (1.7)	7 (11.7)		8 (6.7)	
Quantitative variables (unit)						
		Case (n=60)		Control (n=60)	Total (Mean±SD)	p-value ^b^
Age (year)		56.78±12.31		63.75±12.71	60.29±12.94	0.003
BMI (kg/m^2^)		27.31±3.95		25.87±5.82	26.59±5	0.12
HR (/min)		81.88±15.85		83.44±19.62	82.65±17.74	0.63
Body Temperature (°C)		36.74±0.27		36.75±0.39	36.74±0.33	0.79
RR (/min)		24.68±3.98		26.49±13.35	25.56±9.74	0.07
WBC (x 10^9^/L)		11.7±7.39		13.2±7.52	12.46±7.46	0.27
Systolic BP (mmHg)		123.73±20.43		118.66±19.03	121.2±19.82	0.16
Diastolic BP (mmHg)		73.51±16.46		74.06±20.22	73.79±18.36	0.87
LYM (x 10^9^/L)		6.4 (5.1, 9)		6.65(4.22,9.37)	6.6 (4.75,9.05)	0.34
NEUT (x 10^9^/L)		86.45±6.6		87.21±6.52	86.81±7.66	0.66
Serum Creatinine (mg/dL)		1.1 (0.9, 1.3)		1.2 (0.9, 1.7)	1.1 (0.9, 1.5)	0.11
SPO_2_ (%)		89.68±7.69		86.66±10.07	88.19±9.03	0.07
SGOT (U/L)		44 (34, 55)		39 (25, 71)	40.5 (27.75,62.5)	0.76
SGPT (U/L)		42 (30.75, 70.5)		39 (28, 68)	40 (30, 68.5)	0.55
Hb (g/dL)		13.84±2.25		13.76±2.46	13.8±2.35	0.85
pH		7.32±.78		7.36±0.1	7.34±0.55	0.69
PCO_2_ (mmHg)		41.42±9.76		42.99±11.93	42.21±10.88	0.43
Total Bilirubin (mg/dL)		.84±.44		1.24±1.83	0.89±0.5	0.21
INR		1.24±.28		1.22±0.3	1.23±0.29	0.72
CRP (μg/ml)		51.68±27.18		54.48±26.97	52.83±26.99	0.61
LDH (U/L)		1096.8±393.05		1199±730.33	1144.63±574.32	0.36
Pulmonary involvement (%)		68.53±9.6		70.37±8.87	69.07±9.37	0.42
Sodium (mmol/L)		137.7±3.98		137.9±8,72	137.8±6.75	0.87
Potassium (mmol/L)		4.13±0.49		4.31±.,85	4.22±0.69	0.16
Calcium (mg/dL)		8.36±74		8.08 v 1.15	8.22±0.97	0.13
Phosphorus (mg/dL)		3.29±0.79		3.5±1.52	3.38±1.16	0.41
Magnesium (mg/dL)		2.43±.38		2.49±.87	2.42±0.42	0.65

Note: Data are mean± SD, median (IQR), or N (%) as indicated. a Obtained from Chi-square or Fisher exact test b Obtained from independent samples t test (for parametric data) or Mann–Whitney U test (for nonparametric data). BMI: body mass index, HR: heart rate, RR: respiratory rate, WBC: white blood cells, LYM: lymphocyte, NEUT: neutrophil, BP: blood pressure, INR: international normalized ratio, SGOT: serum Glutamic-oxalacetic transaminase, SGPT: serum glutamic-pyruvic transaminase (SGPT), LDH: lactate dehydrogenase, Hb: hemoglobin

**Primary outcomes**

**Mortality**

**Figure 2:**
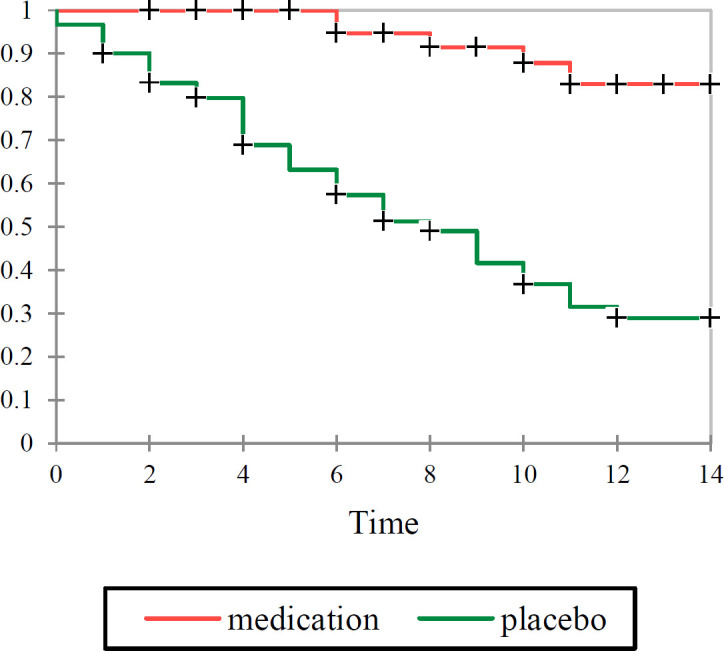
Kaplan-Meier survival analysis

Until day 14, death occurred in five patients (8.33%) in the antiviral preparation group, while in 36 patients (60%) of the control group, which means highly significant difference in mortality between medication and placebo groups. The probability of death for patients who received the medication was 0.14 times of those for the placebo group (risk ratio: 0.14; 95% confidence interval [CI], 0.05 to 0.32; p<0.001). To determine the odds ratio of mortality, logistic regression analysis was conducted. The results demonstrated that the age-adjusted odds ratio of death for the placebo group compared to the antiviral preparation group was 14.63 (95% confidence interval [CI], 4.95 to 43.24; p<0.001). This model adequately fit the data according to the Hosmer-Lemeshow goodness-of-fit test (statistic =7.05, p=0.53). We also evaluated the effect of the intervention on median survival during 14 days as age-adjusted hazard ratio derived from Cox regression was 0.12 (95% confidence interval [CI], 0.04 to 0.31; p<0.001). The Kaplan-Meier curve along with Log Rang test also showed significant difference in the survival rate between the groups (Figure 2). In fact, the patients who received the antiviral preparation were predicted with higher survival compared to the placebo group.

**Secondary outcomes**

Due to low sample size of the placebo group resulting from missed dead patients on the 14^th^ day, improvement in several clinical and laboratory parameters of patients was evaluated through day 7 (Table 2). Some respiratory parameters like respiratory rate (RR) showed significant improvement in patients treated with the antiviral preparation on day 7 compared to the baseline measurement (difference mean: -2.22±3.37, p=0.001); however, the change was not significant compared to the placebo group (difference mean: -0.73±5.82, p=0.53). Several laboratory parameters like peripheral blood lymphocyte (difference median: 3.1(-0.17, 6.25), p=0.007) and neutrophil count (difference mean: -6.22±8.62, p=0.003), and total bilirubin (difference mean: 0.25±0.59, p=0.02) showed improvement in the antiviral preparation-treated patients, while lymphocyte count (difference median: 1.6 (-0.77, 3.62), p=0.04) and serum glutamic pyruvic transaminase (SGPT) (difference median: 12.5(-0.5, 53.25), p=0.03) were exacerbated in the placebo group. Overall, white blood cells (WBCs) count [case: -0.12±7.65, p=0.92 vs. control: -3.11±9.2, p=0.08], CRP [case: -20(-45, -7.5), p<0.001 vs. control: 0(-0.26, 10), p=0.47], and calcium [case: 0.4±0.95, p=0.02 vs control: 0.17±1.4, p=0.53] were improved in antiviral preparation-treated patients compared to the placebo group. Importantly, SpO_2_ [case: 3.02±7.93, p=0.005 vs control: -3.8±12.83), p=0.07] demonstrated highly significant improvement in the treatment compared with the placebo group. No adverse effect was reported for patients in association with the treatment.

**Table 2:** Clinical and laboratory variables post intervention

Variables (Unit)	groups	Day 0	Day 7	p-value ^a^ (Within group)	p-value ^b^ (Between group)
HR (/min)	Medication	82.03±17.84	85.2±13.84	0.28	0.05
	Placebo	87.44±16.45	93.32±13.67	0.13	
Body temperature (ºC)	Medication	36.71±0.28	36.7±0.32	0.86	0.32
	Placebo	36.75±0.24	36.8±0.36	0.41	
RR (/min)	Medication	24.93±3.55	22.71±3.26	0.001	0.53
	Placebo	23.8±5.49	23.07±2.93	0.52	
WBC (x 10^9^/L)	Medication	12.4±9.49	12.2±4.84	0.92	0.05
	Placebo	12.45±6.33	15.56±7.55	0.08	
Systolic BP (mmHg)	Medication	125.69±21.54	124.54±15.48	0.66	0.18
	Placebo	118.48±18.77	116.76±14.75	0.69	
Diastolic BP (mmHg)	Medication	74.21±12.83	75.03±11.41	0.73	0.69
	Placebo	77.11±28.75	73.64±13.7	0.56	
LYM (x 10^9^/L)	Medication	9.12±10.31 6.15 (5.15, 9)	12.49±12.14 8.7 (5.95, 16.37)	0.007	0.31
	Placebo	7.14±4.01 5.6 (4.6, 9)	8.9 ± 5.11 7.1 (5.5, 12.85)	0.04	
NEUT (x 10^9^/L)	Medication	85.64±10.95	79.42±13.69	0.003	0.28
	Placebo	87.9±5.42	84.51±7.73	0.03	
Serum creatinine (mg/dL)	Medication	1.1 (0.9, 1.4)	1.2 (0.9, 1.4)	0.93	0.58
	Placebo	1.2 (0.9, 1.45)	1.05 (0.8, 1.3)	0.21	
SpO_2_ (%)	Medication	89.68±7.69	92.7±3.39	0.005	<0.001
	Placebo	87.54±10.23	83.74±13.49	0.07	
SGOT (U/L)	Medication	44 (34, 55)	43 (30, 54)	0.35	0.32
	Placebo	38.5 (24.75, 65)	39 (23.75, 90)	0.3	
SGPT (U/L)	Medication	37 (29.75, 58.25)	57 (35.25, 75)	0.09	0.82
	Placebo	36.5 (29.25, 76)	60.5 (42, 89.5)	0.03	
Hb (g/dL)	Medication	13.52±2.4	13.38±2.05	0.61	0.32
	Placebo	14±2.46	13.28±2.88	0.1	
pH	Medication	7.22±1.06	7.23±1.06	0.6	0.07
	Placebo	7.37±0.1	7.15±0.78	0.12	
PCO_2_ (mmHg)	Medication	41.44±10.71	43±9.21	0.36	0.44
	Placebo	40.75±9.3	45.88±14.02	0.06	
Total bilirubin (mg/dL)	Medication	0.9±0.55	1.16±0.7	0.02	0.81
	Placebo	0.87±0.33	1.1±0.42	0.08	
INR	Medication	1.23±0.23	1.14±0.23	0.1	0.17
	Placebo	1.15±0.18	1.22±0.29	0.29	
CRP (μg/ml)	Medication	53 (27, 72.75)	15 (5.5, 37)	<0.001	0.01
	Placebo	58 (50, 73)	60 (23, 76)	0.47	
LDH (U/L)	Medication	1161.73±440.7	1015.56±451.82	0.06	0.99
	Placebo	1129.76±447.36	999.82±447.36	0.44	
Sodium (mmol/L)	Medication	137.6±4.19	137.18±3.8	0.62	0.83
	Placebo	139.27±12.39	138.15±6.39	0.58	
Potassium (mmol/L)	Medication	4.21±0.53	4.27±0.48	0.61	0.42
	Placebo	4.28±0.81	4.11±0.68	0.49	
Calcium (mg/dL)	Medication	8.18±0.83	8.59±0.65	0.02	0.031
	Placebo	8.08±0.99	8.25±0.75	0.53	
Phosphorus (mg/dL)	Medication	3.44±0.83	3.52±1.15	0.74	0.81
	Placebo	3.39±1.48	3.32±0.99	0.82	
Magnesium (mg/dL)	Medication	2.36±0.33	2.31±0.28	0.44	0.54
	Placebo	2.45±0.42	2.4±0.49	0.7	

## Discussion

The world has been facing the COVID-19 pandemic since late 2019, which has affected millions of lives and threatened billion others. SARS-CoV-2 affects both genders, a wide age range (although with lower pathogenesis in children compared to more severe disease in the elderly), races, and particularly individuals with comorbidities like diabetes, hypertension, and underlying pulmonary diseases in all continents. To date, more than 7 million cases have been diagnosed with COVID-19 among 50 million qRT-PCR tests, and over 140,000 recorded deaths are reported in Iran that shows improvements compared to the first months of the disease outbreak (see https://www.worldometers.info/). The country has experienced several peaks of COVID-19 and high incidence of deaths. That was due to shortages in diagnostic tests available for the population, hospital beds and mechanical ventilation machines, access to vaccines, economic situation of the country, and policies imposed by the policy makers (Ahmadi et al., 2021). Nowadays, COVID-19 load is improving which is suggested to be due to mass vaccinations, preventive strategies, policies upon lockdown of non-essential settings, etc. The majority of COVID-19 cases are asymptomatic, recover from mild illness spontaneously or after receiving supportive therapies. However, millions of deaths have resulted from severe pulmonary involvement contributing to multi-organ failure. The highest rates of mortality among hospitalized patients (up to 88%) have been reported in ICU-admitted cases, which require intensive pulmonary support due to advanced lung involvement and co-morbidities (Rieg et al., 2020). 

Taking less than one year, SARS-CoV-2 vaccines have been developed most quickly compared to any other vaccine in the history (Funk et al., 2020). To date, about 50 vaccines have received approval for clinical use, a number of others are in the pipeline, and more than 70% of the global population in have received at least one dose of a COVID-19 vaccine by mid-October 2023 (Mathieu al., 2020). Globally, these vaccines have shown robust effects in the prevention of severe disease and subsequently reduced COVID-19-associated mortality, particularly among older and susceptible individuals (Dagan et al., 2021; Lopez Bernal et al., 2021; Tregoning et al., 2021). However, new SARS-CoV-2 infections, although mainly milder, have been reported to occur in a number of vaccinated people (Bergwerk et al., 2021). Moreover, to achieve high protection in a society, the majority of the population are required to get vaccinated since unvaccinated individuals may drive further outbreaks (Dhama et al., 2021). This may take more extended time, investment, and comprehensive policies to support mass vaccinations in addition to requirements for global equal distribution. Additionally, high effectiveness of SARS-CoV-2 vaccines against novel viral variants resulted from frequent mutations is a place of doubt and requires further investigations and optimizations (Madhi et al., 2021). Due to lack of definite preventive or therapeutic approaches, conventional interventions like face masks and social distancing are still recommended as non-pharmacological interventions particularly in settings with high possibility of transmission in addition to vaccination for breaking the virus transmission cycle until its eradication (Rowan and Moral, 2021). 

Hitherto, no efficient therapy has been developed for COVID-19 patients and several suggested treatments like convalescent plasma taken from already infected patients, and repurposed drugs like chloroquine, hydroxychloroquine, ribavirin, lopinavir-ritonavir, favipiravir, and ivermectin have failed in several clinical trials to demonstrate significant impact on the course of the disease (Agarwal et al., 2020; López-Medina et al., 2021; Martinez, 2021). Although studies have shown reduced hospitalization, recovery time and mortality with the use of a number of drugs like the antiviral remdesivir (during the early phase of disease), interferons, and dexamethasone (Welte et al., 2021), these agents could not be considered definite treatments for COVID-19 as their efficacies in other studies have been questioned and several drug agencies have not approved their clinical use unlike others (Oliver et al., 2022). Additionally, although mass vaccinations have helped to improve management of patients preventing severe illness, still a fraction of patients are admitted to take intensive support, among them mortality happens particularly in patients with underlying diseases. 

In this randomized, double-blind, placebo-controlled phase III trial, we showed the efficacy and safety of a novel antiviral preparation in ICU-hospitalized patients with COVID-19. In our previous study, we showed that the antiviral preparation inhibited viral load of RNA viruses (data not published yet). Our results demonstrated 91.67% recovery of patients after up to two weeks of treatment, and 8.33% mortality rate in the antiviral preparation-treated patients compared with 40% recovery and 60% mortality in the placebo group. These numbers unveiled a highly significant impact of the antiviral preparation in reducing COVID-19-associated deaths. COVID-19 patients in the control group, who received normal saline in addition to standard care, had age-adjusted odds ratio (OR) of death of about 14.63 compared with those given the antiviral preparation. Survival analysis also demonstrated a significantly higher survival rate in the antiviral preparation-treated *versus* control patients. Additionally, clinical and laboratory parameters demonstrated improvements in the treatment group after 7 days compared with placebo-prescribed patients. The observed efficacy could be attributed to the activity of the herbal ingredients of the preparation against various types of human viral pathogens like hepatitis viruses, attenuated human immunodeficiency virus type 1 (HIV-1), influenza virus, and SARS-CoV (Ito et al., 1987; Sato et al., 1996; Utsunomiya et al., 1997). For instance, *Glycyrrhiza glabra* and its phytochemicals seem to inhibit viral replication, and affect the intracellular signaling, and production of nitric oxide through regulation of inducible nitric oxide synthase in the macrophages (Nassiri Asl and Hosseinzadeh, 2007). It is also known to inhibit SARS-CoV-2 replication *in vitro* via inhibiting the viral main protease M^pro ^(van de Sand et al., 2021). *Allium sativum *L. with Iranian native origin has been reported with activity against the avian coronavirus, antiviral function, and fungistatic effects (Mohajer Shojai et al., 2016; Pontin et al., 2015). Garlic active substances also have shown inhibitory effects on the host receptor angiotensin-converting enzyme 2 (ACE2) protein *in silico* suggested as a prophylactic agent against SARS-CoV-2 (Thuy et al., 2020). Crude polysaccharides extracted from *Syzygium aromaticum* also have shown inhibitory effects on SARS-CoV-2 through blocking its proteases (Vicidomini et al., 2021). Although vaccination is still the best form of prevention, many countries (particularly some in Africa) still have a vaccination rate of less than 10% (https://ourworldindata.org/covid-vaccinations). It is suggested that the use of this natural mixture could aid recovery in such poorly vaccinated countries and territories. The novel preparation introduced in this study may also improve outcomes in those who have been fully vaccinated but this have to be tested in future trials. 

The herbal antiviral preparation demonstrated high efficacy in the treatment of critically ill ICU-admitted patients with COVID-19. It significantly reduced mortality rate, and improved clinical and laboratory parameters compared to the placebo group. Our results demonstrated good efficacy and safety of the preparation surpassing other suggested treatments. Taken together, the novel antiviral preparation can be considered as a potential therapeutic agent for SARS-CoV-2-infected patients, not only for critically ill patients but also for outpatients. Potentially the greatest impact of the use of this preparation will be seen in the likely advent of future pandemics before new vaccines can be produced and deployed around the world. The major limitations of the current study include that it was conducted in a fraction of affected COVID-19 patients and in a localized center and among critically ill patients. Further investigations are recommended to discover the therapeutic effects of the antiviral preparation in larger populations and multicenter trials inclusive of different ethnicities and age groups. Given the safety of the preparation, it is also recommended that different subsets of COVID-19 patients (*e.g.,* outpatients and hospitalized ones) be studied. Finally, pharmacological studies are suggested to unravel the mechanisms underlying the therapeutic effects of this herbal preparation.

## References

[B1] Agarwal A, Mukherjee A, Kumar G, Chatterjee P, Bhatnagar T, Malhotra P (2020). Convalescent plasma in the management of moderate covid-19 in adults in India: open label phase II multicentre randomised controlled trial (PLACID Trial). BMJ.

[B2] Ahmadi ZH, Mousavizadeh M, Nikpajouh A, Bahsir M, Hosseini S (2021). COVID-19: A perspective from Iran. J Card Surg.

[B3] Arentz M, Yim E, Klaff L, Lokhandwala S, Riedo FX, Chong M, Lee M (2020). Characteristics and outcomes of 21 critically Ill patients with COVID-19 in Washington State. JAMA.

[B4] Bayat M, Asemani Y, Najafi S (2021). Essential considerations during vaccine design against COVID-19 and review of pioneering vaccine candidate platforms. Int Immunopharmacol.

[B5] Bergwerk M, Gonen T, Lustig Y, Amit S, Lipsitch M, Cohen C, Mandelboim M, Levin EG, Rubin C, Indenbaum V, Tal I, Zavitan M, Zuckerman N, Bar-Chaim A, Kreiss Y, Regev-Yochay G (2021). Covid-19 Breakthrough Infections in Vaccinated Health Care Workers. N Engl J Med.

[B6] Dagan N, Barda N, Kepten E, Miron O, Perchik S, Katz MA, Hernán MA, Lipsitch M, Reis B, Balicer RD (2021). BNT162b2 mRNA Covid-19 Vaccine in a Nationwide Mass Vaccination Setting. N Engl J Med.

[B7] Dhama K, Sharun K, Tiwari R, Dhawan M, Emran TB, Rabaan AA, Alhumaid S (2021). COVID-19 vaccine hesitancy – reasons and solutions to achieve a successful global vaccination campaign to tackle the ongoing pandemic. Hum Vaccin Immunother.

[B8] Elezkurtaj S, Greuel S, Ihlow J, Michaelis EG, Bischoff P, Kunze CA, Sinn BV, Gerhold M, Hauptmann K, Ingold-Heppner B, Miller F, Herbst H, Corman VM, Martin H, Radbruch H, Heppner FL, Horst D (2021). Causes of death and comorbidities in hospitalized patients with COVID-19. Sci Rep.

[B9] Funk CD, Laferrière C, Ardakani A (2020). A Snapshot of the Global Race for Vaccines Targeting SARS-CoV-2 and the COVID-19 Pandemic. Front Pharmacol.

[B10] Hu S, Wang W, Wang Y, Litvinova M, Luo K, Ren L, Sun Q, Chen X, Zeng G, Li J, Liang L, Deng Z, Zheng W, Li M, Yang H, Guo J, Wang K, Chen X, Liu Z, Yan H, Shi H, Chen Z, Zhou Y, Sun K, Vespignani A, Viboud C, Gao L, Ajelli M, Yu H (2021). Infectivity, susceptibility, and risk factors associated with SARS-CoV-2 transmission under intensive contact tracing in Hunan, China. Nat Commun.

[B11] Ito M, Nakashima H, Baba M, Pauwels R, De Clercq E, Shigeta S, Yamamoto N (1987). Inhibitory effect of glycyrrhizin on the in vitro infectivity and cytopathic activity of the human immunodeficiency virus [HIV (HTLV-III/LAV)]. Antiviral Res.

[B12] López-Medina E, López P, Hurtado IC, Dávalos DM, Ramirez O, Martínez E, Díazgranados JA, Oñate JM, Chavarriaga H, Herrera S, Parra B, Libreros G, Jaramillo R, Avendaño AC, Toro DF, Torres M, Lesmes MC, Rios CA, Caicedo I (2021). Effect of Ivermectin on time to resolution of symptoms among adults with mild COVID-19: A randomized clinical trial. JAMA.

[B13] Lopez Bernal J, Andrews N, Gower C, Robertson C, Stowe J, Tessier E, Simmons R, Cottrell S, Roberts R, O'Doherty M, Brown K, Cameron C, Stockton D, McMenamin J, Ramsay M (2021). Effectiveness of the Pfizer-BioNTech and Oxford-AstraZeneca vaccines on covid-19 related symptoms, hospital admissions, and mortality in older adults in England: test negative case-control study. BMJ.

[B14] MacLaren G, Fisher D, Brodie D (2022). Treating the most critically Ill patients with COVID-19: The evolving role of extracorporeal membrane oxygenation. JAMA.

[B15] Madhi SA, Baillie V, Cutland CL, Voysey M, Koen AL, Fairlie L, Padayachee SD, Dheda K, Barnabas SL, Bhorat QE, Briner C, Kwatra G, Ahmed K, Aley P, Bhikha S, Bhiman JN, Bhorat AE, du Plessis J, Esmail A, Groenewald M, Horne E, Hwa SH, Jose A, Lambe T, Laubscher M, Malahleha M, Masenya M, Masilela M, McKenzie S, Molapo K, Moultrie A, Oelofse S, Patel F, Pillay S, Rhead S, Rodel H, Rossouw L, Taoushanis C, Tegally H, Thombrayil A, van Eck S, Wibmer CK, Durham NM, Kelly EJ, Villafana TL, Gilbert S, Pollard AJ, de Oliveira T, Moore PL, Sigal A, Izu A (2021). Efficacy of the ChAdOx1 nCoV-19 covid-19 vaccine against the B. 1.351 variant. N Engl J Med.

[B16] Martinez MA (2021). Lack of effectiveness of repurposed drugs for COVID-19 treatment. Front Immunol.

[B17] Mathieu E, Ritchie H, Ortiz-Ospina E, Roser M, Hasell J, Appel C, Giattino C, Rodés-Guirao L (2021). A global database of COVID-19 vaccinations. Nat Hum Behav.

[B18] Mohajer Shojai T, Ghalyanchi Langeroudi A, Karimi V, Barin A, Sadri N (2016). The effect of Allium sativum (Garlic) extract on infectious bronchitis virus in specific pathogen free embryonic egg. Avicenna J Phytomed.

[B19] Myers LC, Parodi SM, Escobar GJ, Liu VX (2020). Characteristics of hospitalized adults with COVID-19 in an integrated health care system in California. JAMA.

[B20] Nassiri Asl M, Hosseinzadeh H (2007). Review of Antiviral Effects of Glycyrrhiza glabra L and Its Active Component, Glycyrrhizin. J Med Plant Res.

[B21] Oliveira E, Parikh A, Lopez-Ruiz A, Carrilo M, Goldberg J, Cearras M, Fernainy K, Andersen S, Mercado L, Guan J, Zafar H, Louzon P, Carr A, Baloch N, Pratley R, Silverstry S, Hsu V, Sniffen J, Herrera V, Finkler N (2021). ICU outcomes and survival in patients with severe COVID-19 in the largest health care system in central Florida. PLoS One.

[B22] Oliver JC, Silva EN, Soares LM, Scodeler GC, Santos AS, Corsetti PP, Prudêncio CR, de Almeida LA (2022). Different drug approaches to COVID-19 treatment worldwide: an update of new drugs and drugs repositioning to fight against the novel coronavirus. Ther Adv Vaccines Immunother.

[B23] Piroth L, Cottenet J, Mariet AS, Bonniaud P, Blot M, Tubert-Bitter P, Quantin C (2021). Comparison of the characteristics, morbidity, and mortality of COVID-19 and seasonal influenza: a nationwide, population-based retrospective cohort study. Lancet Respir Med.

[B24] Pontin M, Bottini R, Burba JL, Piccoli P (2015). Allium sativum produces terpenes with fungistatic properties in response to infection with Sclerotium cepivorum. Phytochemistry.

[B25] Rieg S, von Cube M, Kalbhenn J, Utzolino S, Pernice K, Bechet L, Baur J, Lang CN, Wagner D, Wolkewitz M, Kern WV, Biever P (2020). COVID-19 in-hospital mortality and mode of death in a dynamic and non-restricted tertiary care model in Germany. PLoS One.

[B26] Rowan NJ, Moral RA (2021). Disposable face masks and reusable face coverings as non-pharmaceutical interventions (NPIs) to prevent transmission of SARS-CoV-2 variants that cause coronavirus disease (COVID-19): Role of new sustainable NPI design innovations and predictive mathematical modelling. Sci Total Environ.

[B27] Sato H, Goto W, Yamamura J, Kurokawa M, Kageyama S, Takahara T, Watanabe A, Shiraki K (1996). Therapeutic basis of glycyrrhizin on chronic hepatitis B. Antiviral Res.

[B28] Thuy BTP, My TTA, Hai NTT, Hieu LT, Hoa TT, Thi Phuong Loan H, Triet NT, Anh TTV, Quy PT, Tat PV, Hue NV, Quang DT, Trung NT, Tung VT, Huynh LK, Nhung NTA (2020). Investigation into SARS-CoV-2 resistance of compounds in garlic essential oil. ACS Omega.

[B30] Utsunomiya T, Kobayashi M, Pollard RB, Suzuki F (1997). Glycyrrhizin, an active component of licorice roots, reduces morbidity and mortality of mice infected with lethal doses of influenza virus. Antimicrob Agents Chemother.

[B31] van de Sand L, Bormann M, Alt M, Schipper L, Heilingloh CS, Steinmann E, Todt D, Dittmer U, Elsner C, Witzke O, Krawczyk A (2021). Glycyrrhizin effectively inhibits SARS-CoV-2 replication by inhibiting the viral main protease. Viruses.

[B32] Vicidomini C, Roviello V, Roviello GN (2021). Molecular Basis of the Therapeutical Potential of Clove (Syzygium aromaticum L ) and Clues to Its Anti-COVID-19 Utility. Molecules.

[B33] Wang C, Horby PW, Hayden FG, Gao GF (2020). A novel coronavirus outbreak of global health concern. Lancet.

[B34] Welte T, Ambrose LJ, Sibbring GC, Sheikh S, Müllerová H, Sabir I (2021). Current evidence for COVID-19 therapies: a systematic literature review. Eur Respir Rev.

